# Circulating MIC-1/GDF15 is a complementary screening biomarker with CEA and correlates with liver metastasis and poor survival in colorectal cancer

**DOI:** 10.18632/oncotarget.15279

**Published:** 2017-02-11

**Authors:** Xiaobing Wang, Zhaogang Yang, Haimei Tian, Yanfen Li, Mo Li, Wenya Zhao, Chao Zhang, Teng Wang, Jing Liu, Aili Zhang, Di Shen, Cuining Zheng, Jun Qi, Dan Zhao, Junfeng Shi, Liliang Jin, Jianyu Rao, Wei Zhang

**Affiliations:** ^1^ Tumor Marker Research Center, Cancer Institute and Hospital, Chinese Academy of Medical Sciences and Peking Union Medical College, Beijing, PR China; ^2^ NSF Nanoscale Science and Engineering Center (NSEC), The Ohio State University, Columbus, OH, USA; ^3^ Laboratory of Clinical Biochemistry, Cancer Institute and Hospital, Chinese Academy of Medical Sciences and Peking Union Medical College, Beijing, PR China; ^4^ Department of Gynecological Oncology, Cancer Institute and Hospital, Chinese Academy of Medical Sciences and Peking Union Medical College, Beijing, PR China; ^5^ Department of Mechanical Engineering, The Ohio State University, Columbus, OH, USA; ^6^ Department of Pathobiological Sciences, Louisiana State University, Baton Rouge, LA, USA

**Keywords:** colorectal cancer, biomarker, screening, liver metastasis, prognosis

## Abstract

Macrophage inhibitory cytokine 1 (MIC-1/GDF15) has been characterized as a candidate biomarker for colorectal cancer (CRC) recently. However, the role of serum MIC-1 in screening patients with early stage CRC and monitoring therapeutic response have not been well-established, particularly in the combination with CEA for the screening and the prejudgment of occurrence with liver metastasis. In this study, we performed a retrospective blinded evaluation of 987 serum samples from 473 individuals with CRC, 25 with adenomatous polyps, and 489 healthy individuals using ELISA or immunoassay. The sensitivity of serum MIC-1 was 43.8% and 38.5% for CRC diagnosis and early diagnosis, respectively, which were independent of and comparatively higher than for CEA (36.6% and 27.3%) at comparable specificity. Serum MIC-1 after surgery were significantly elevated at the time of tumor recurrence, and notable increase were observed in 100% patients with liver metastasis. Besides the TNM classification and differentiation grade, MIC-1 was an independent prognostic factor contributing to overall survival. We conclude that MIC-1 can act as a candidate complementary biomarker for screening early-stage CRC by combination with CEA, and furthermore, for the first time, identify a promising prognostic indicator for monitoring recurrence with liver metastasis, to support strategies towards personalized therapy.

## INTRODUCTION

Colorectal cancer (CRC) is the third most prevalent cancer and the fourth leading cause of cancer-related deaths worldwide [1]. In 2008, more than 1 million people were newly diagnosed and over 600,000 patients died from the disease [2]. Owning to its slow development from removable precancerous lesions and curable early stages, screening for CRC in the high-risk population has the utmost potential to reduce the mortality of the disease [3]. Unfortunately, the most reliable invasive colonoscopy, and the currently most widely used noninvasive fecal occult blood test (FOBT), have inconvenience and low sensitivity limitations [4–6]. Blood testing for CRC screening is more compliant and acceptable [7]; however, no specific molecular biomarkers have been identified and validated so far that allow reliably for an accurate diagnosis of CRC. The search for novel biomarkers based on the analysis of blood samples has become a trend of current research.

Carcinoembryonic antigen (CEA) has been used as a serum biomarker for CRC diagnosis and prognosis for several years, and its significance and usefulness in clinical applications have been reported in many studies [8, 9]. However, CEA detection has its limitation, including its relatively low sensitivity and specificity, which make it insufficient for screening large asymptomatic patients alone, and its efficacy for predicting prognosis and monitoring CRC patients remains controversial [10, 11]. Therefore, the discovery and validation of novel biomarkers for CRC would be of utmost clinical importance in routine healthcare for general population and postoperative surveillance for patients undergo surgery.

Recently Macrophage inhibitory cytokine 1 (MIC-1/GDF15) has been explored as a candidate tumor marker for CRC [12, 13]. MIC-1 is a 25-kDa secreted protein of transforming growth factor-β (TGF-β) super-family that has been shown to play an important role in carcinogenesis related activities, including proliferation, migration, apoptosis, and angiogenesis, as well as to be involved in abnormal immune response [14–16]. The discovery of this circulating inflammation markers prospectively associated with CRC could aid in identifying individuals at highest CRC risk. Many studies reported serum MIC-1 as a promising tumor marker of CRC and MIC-1 levels were closely correlated with outcome [12, 13, 17–19]. However, a comprehensive *in vivo* confirmation of MIC-1 in the screening and monitoring for patients with CRC, particularly in the prejudgment of liver metastasis, remains pending. In this study, we systematically evaluate MIC-1 as a candidate complementary biomarker for screening early-stage CRC by comparison with CEA, and furthermore, for the first time, identifying a promising prognostic indicator for monitoring recurrence with liver metastasis, to support strategies towards personalized therapy.

## RESULTS

### The elevated level of serum MIC-1 and its diagnostic efficacy in CRC

**Figure 1:**
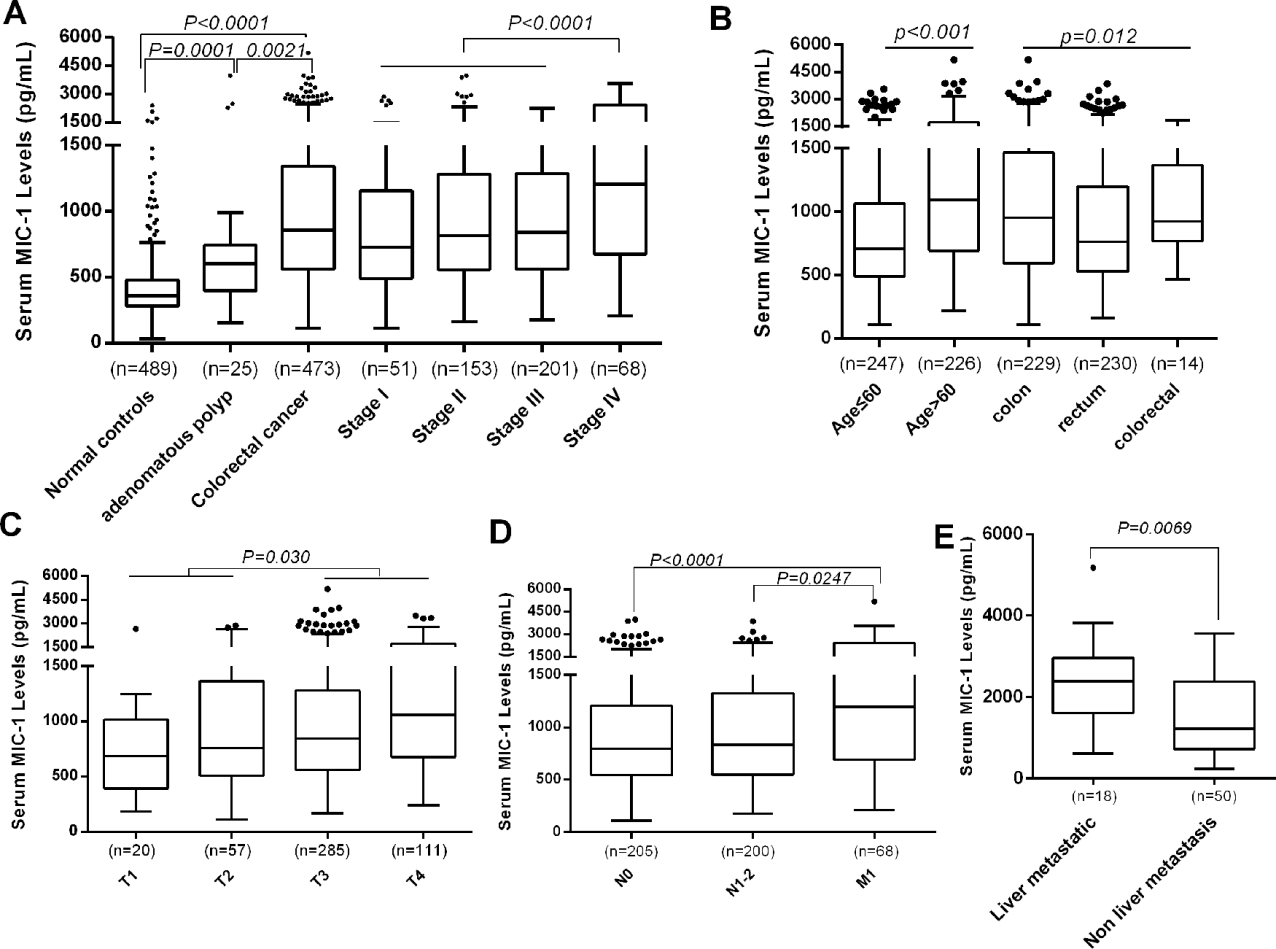
The level of serum MIC-1 in patients with CRC and control **A**. The level of serum MIC-1 in patients with CRC was compared with benign disease and healthy controls. Serum MIC-1 in patients with CRC is significantly higher than that in others (P<0.0001). And the gradual elevation in serum MIC-1 was clearly discernible, with significantly higher serum level in stage IV than in stage I–III (P<0.0001). **B-D**. The level of serum MIC-1 was compared between different clinical characters in the patients with CRC. The level of serum MIC-1 was significantly higher in patients with colon carcinoma and old age (B), depth of tumor invasion (C) and remote metastasis (D). **E**. The level of serum MIC-1 in CRC patients with liver metastasis was compared with other organ metastasis at time of diagnosis. MIC-1 levels are significantly higher in patients with liver metastasis (P=0.0069). In the box plots listed with Tukey's method, the lines represent 10th, 25th, median, 75th and 90th percentiles for each, and the data was statistically calculated using the Mann–Whitney U test.

A stepwise increased serum MIC-1 levels in patients with benign conditions (median, 603.6 pg/mL; range, 154.8-3975.8 pg/mL; P=0.0001) and CRC (median, 859.2pg/mL; range, 112.0-5178.1 pg/mL; P<0.0001) in comparison with healthy control subjects (median, 359.9 pg/mL; range, 33.9-2398.9 pg/mL) were notably observed (Figure 1A). Furthermore, when all patients with CRC were subdivided according to tumor stage, the gradual increase in serum MIC-1 levels was clearly discernible (P=0.0001), with significantly higher concentrations in stage IV than in stage I–III (P<0.0001), implying that the increased serum MIC-1 might have the positive correlation with occurrence and remote metastasis of CRC. Additionally, there was significant association between the level of serum MIC-1 with primary tumor site and age, respectively, with higher level in patients with colon carcinoma and >60 age (P=0.012; P<0.0001; Figure 1B). However, no statistical association between the level of serum MIC-1 with sex, tumor differentiation, and pathological type was observed, respectively.

Further analysis showed that the serum MIC-1 in the T_3-4_ stage group was significantly higher than that in T_1-2_ group (p=0.030; Figure 1C). The results also indicated that MIC-1 level of M_1_ was significantly higher than that in N_1-2_ and N_0_ group (p=0.0247, p<0.0001; Figure 1D); and strikingly, serum levels of MIC-1 were higher in patients with liver metastasis (median, 2322.8pg/mL; range, 616.9-5178.1) when compared with other organ metastasis (median, 1236.1pg/mL; range, 240.8-3565.4, P=0.0069; Figure 1E), suggesting increased levels of serum MIC-1 were significantly correlated with local and remote metastasis, especially liver metastasis.

### Better performance of serum MIC-1 compared with CEA in CRC diagnosis

**Figure 2:**
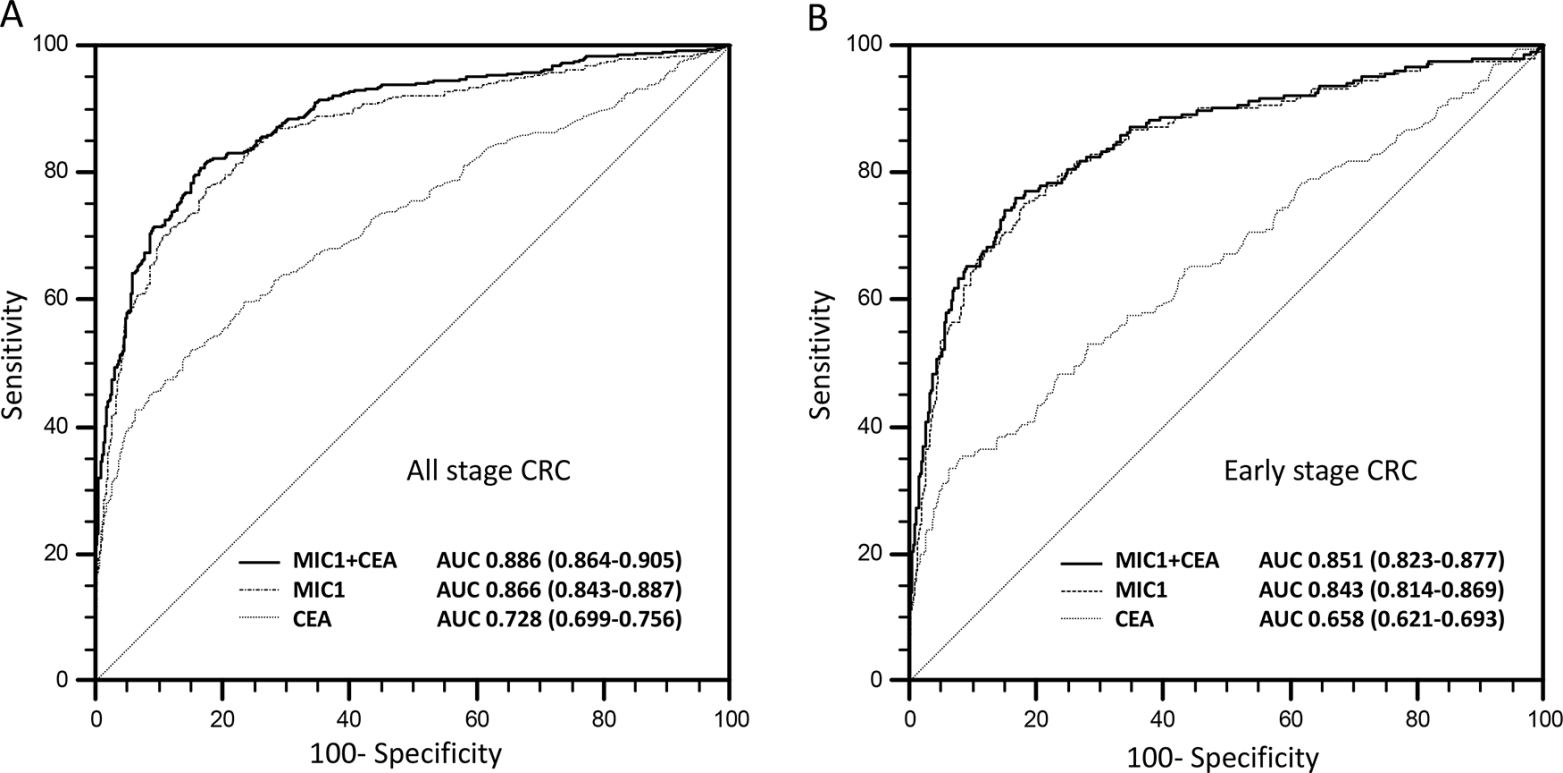
The diagnostic performance of serum MIC-1 and its combination with CEA for CRC **A**. AUROC of serum MIC-1 was higher than that of CEA (P<0.001), and combination of MIC-1 and CEA would enhance the diagnostic performance significantly (P<0.001). **B**. The efficacy of serum MIC-1 in the detection of early stage CRC is significantly higher than CEA. ROC curve analysis showed the combination use of serum MIC-1 and serum CEA for discriminating early stage CRC will be valued in the screening of CRC.

The performance of serum MIC-1 as a non-invasive biomarker for CRC was assessed by generating ROC curves and comparing with CEA. Using the 489 samples from healthy subjects as controls, the area under the ROC curve of MIC-1 (AUC: 0.866, 95%CI: 0.843-0.887) for CRC is higher than that of CEA (AUC: 0.728, 95%CI: 0.699-0.756; P<0.0001; Figure 2A). Using the serum level of 1000 pg/ml MIC-1 as clinical reference value, which calculated by mean value plus three times standard deviations of healthy controls and accounted for the sake of convenient usability in clinical, the sensitivity, specificity, PPV and NPV of MIC-1 were 43.8%, 96.7%, 92.8%, and 64.1%, respectively, to identify patients with CRC. The sensitivity of MIC-1 for diagnosis of CRC was better than that of CEA (43.8% vs 36.6%) and demonstrated comparable specificity (96.7% vs 95.9%), suggesting that MIC-1 can be used as a much more sensitive tumor serum biomarker compared to CEA for the detection of CRC. Moreover, results showed that the sensitivity of serum MIC-1 was independent of serum CEA (χ2=10.439, P=0.0012), indicating the combination of MIC-1 and CEA may enhance the detection of CRC. MIC-1 demonstrated a sensitivity of 47.3% in those CRC with negative CEA (<5 U/mL; n=300) with a median MIC-1 value of 935.4 pg/mL. Moreover, results showed that the combination of MIC-1 and CEA could improve the diagnostic performance significantly (AUROC: 0.886; 95% CI: 0.864-0.905; P=0.0001), at a 72.7% sensitivity and 89.0% specificity (Figure 2A), by multivariate logistic regression model.

To further assess the performance of MIC-1 in early CRC detection and diagnosis, a subgroup of patients with early-stage CRC were evaluated (stage I and II; n=205). The serum MIC-1 (AUC: 0.843, 95%CI: 0.814-0.869) showed a better performance compared with CEA (AUC: 0.658, 95%CI: 0.621-0.693; P<0.0001) for distinguishing early-stage CRC from normal controls (Figure 2B) by ROC curve analysis. Notably, MIC-1 alone can achieve 38.5% (79/205) positive detection rate in early-stage CRC patients (stage I and II) whereas CEA can only detect 27.3% early-stage patients, suggesting that MIC-1 could be used as a potential biomarker for early-stage CRC detection.

### Serum MIC-1 decreased after surgical removal and significantly increased at relapse

**Figure 3:**
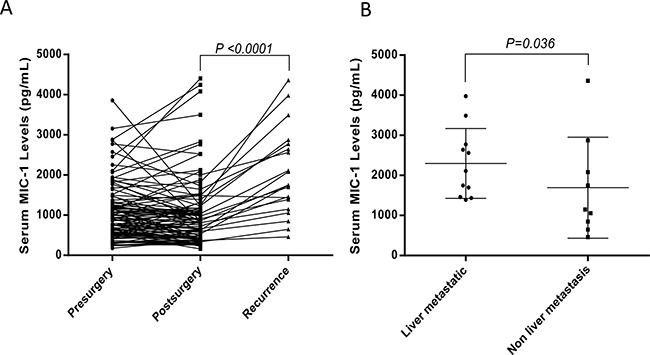
The value of serum MIC-1 in assessment of therapy response and surveillance of CRC recurrence after curative resection **A**. The level of serum MIC-1 in CRC patients before surgery was compared with that of one month after surgical removal of primary tumors (n = 106). And in 20 patients with documented CRC recurrence, the level of serum MIC-1 were significantly elevated (median: 841.2 pg/mL vs 1747.0 pg/mL, mean + SD: 1017+832.1 pg/mL vs 1902+953.5 pg/mL; p<0.001). **B**. The level of serum MIC-1 in the patients with liver metastasis was compared with non-liver metastasis at the presence of tumor recurrence in 20 relapse patients. More highly elevated levels of serum MIC-1 was occurred 100% among 11 patients with liver metastasis, compared with MIC-1 levels in patients with non-liver metastasis (n=9).

4 weeks after surgery, we collected post-operative serum from 106 of those patients who received surgery treatment. Serum MIC-1 levels were only slightly decreased from 1058.6±703.2 pg/mL to 1016.7±832.1 pg/mL (p=0.1853) at 4 weeks after resection. But when serum was detected and analyzed based on 20 relapse patients from above 106 patients, the decreased level of serum MIC-1 after operation (1026.5±494.1 pg/mL) was significantly elevated at the presence of tumor recurrence (1848.6±950.9 pg/mL; P <0.0001) (Figure 3A). These results indicated that MIC-1 could be exploited as a potential serum biomarker to monitor the post-operative recurrence in patients with CRC.

Furthermore, when data was compared between post-operative liver metastasis and other metastasis in relapse patients, statistically significant difference was observed in MIC-1 levels in patients with liver metastasis (n=11) and non-liver metastasis (n=9) (2298.3±871.8 pg/mL vs 1544.1±1259.9 pg/mL; P=0.036) (Figure 3B), but notably, more highly elevated levels of serum MIC-1 was occurred 100% among 11 patients with liver metastasis. Collectively, all these results emphasize the importance of serum MIC-1 as a potential biomarker for surveillance of the early CRC recurrence, especially for liver metastasis.

### Serum MIC-1 negatively correlates with the prognosis of CRC

**Figure 4:**
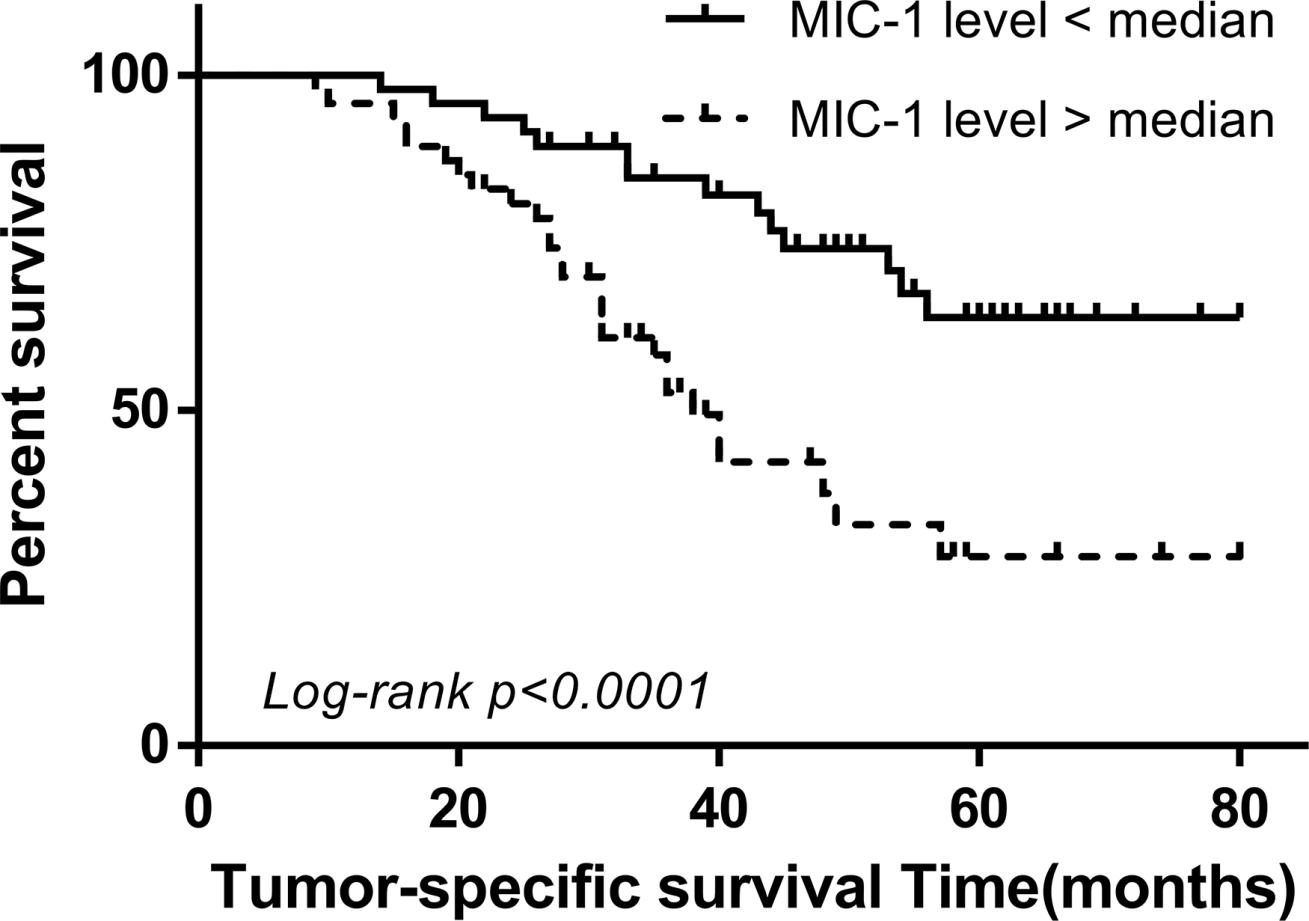
The value of serum MIC-1 in the prediction of CRC prognosis Tumor-specific survival curves were prepared and analyzed between two divided groups according to the median levels of serum MIC-1 in patients before treatment (average, 43 months; range, 9–80 months; n=94). Patients with higher serum MIC-1 had a trend to poorer tumor-specific survival (median survival time: 34 vs 50 months, P<0.0001).

Follow-up data was obtained for 94 patients undergoing surgical resection. Patients were separated into pre-operative low-level and high-level group, using median value as the indicator, to investigate the link between serum MIC-1 and the clinical outcome of CRC patients. A log-rank test showed that patients with higher level of serum MIC-1 had a trend to poorer tumor-specific survival (P=0.0005; Figure 4). A univariate Cox regression analysis on the tumor-specific overall survival was performed and results suggest that the TNM stage, differentiation and serum CEA were also significantly associated. However, gender, age and primary tumor location showed no correlation. To further evaluate whether serum MIC-1 can be used as a prognostic biomarker in CRC patients, regression analysis using the Cox's proportional hazards model was performed. The covariate parameters included several significant clinicpathological factors observed in univariate analysis in addition to MIC-1, as listed in Table 1. The results showed that, besides the TNM stage and differentiation, MIC-1 was an independent prognostic indicator contributing to tumor-specific overall survival after correction for all of these factors [hazard ratio of tumor death: 2.607(95%CI, 1.312-5.181), P=0.007] (Table 1).

**Table 1:** Univariate and multivariate Cox proportional hazard modeling of factors associated with tumor-specific survival in CRC patient group (n = 94)

Variable	Subset	Hazard Ratio	95 % CI	P value
**(a) Univariate analysis by log-rank**				
Age (years)	>60/≤60	1.605	0.845 - 3.049	0.125
Gender	Male/female	0.939	0.503 - 1.751	0.842
Tumor site	Colon/rectum	1.017	0.549 -1.886	0.957
Differentiation grade	Low / High-moderately	3.882	1.705 - 8.837	0.042
TNM stage	III-IV/I-II	3.966	2.148 - 7.322	0.0001
CEA level	High / Low	2.117	1.144 - 3.917	0.017
MIC-1 level	High / Low	2.917	1.561 - 5.452	0.0005
**(b) Multivariate analysis by Cox proportional hazard model**				
Differentiation grade	Low / High-moderately	2.487	1.016-6.091	0.047
TNM stage	III-IV / I-II	3.508	1.636- 7.519	0.001
CEA level	High / Low	1.644	0.844 -3.202	0.146
MIC-1 level	High / Low	2.607	1.312-5.181	0.007

## DISCUSSION

CRC is considered to be one of the most prevalent carcinomas in the world, with about one million new cases and half a million mortalities each year [1, 2]. The identification of a non-invasive test, with an outstanding diagnostic performance and high patient compliance, improves the prognosis of patients and is a key factor to reduce the mortality from CRC [20, 21]. In our present study, we assessed MIC-1 as a serum biomarker for detection of CRC and demonstrated that serum MIC-1 can contribute to improve the performance of CEA for detecting CRC patients, complementing its capacity when offered to CEA negative individuals. Importantly, the value of serum MIC-1 as a prognostic marker in CRC was evaluated, and its ability for tumor recurrence prediction was investigated; and to our knowledge, the present research is the first investigation into the potential clinical value of serum MIC-1 in CRC patients with liver metastasis at diagnosis and post-operation relapse.

First, in the retrospective case–control study reported here, we found that patients with CRC cancer have a much higher MIC-1 level in serum compared with healthy controls as reported before, implying that serum MIC-1 would serve as a potential serum biomarker for differential diagnosis of CRC. In addition, we also discovered that serum MIC-1 in patients with early-stage tumors (Stage I-II) were significantly higher than that in non-patient controls, and the serum levels of MIC-1 are elevated with cancer stage. These results indicate that serum MIC-1 may be increased in the early stage and correspond with progression of CRC. And in our study, MIC-1 showed high sensitivity with 43.8% at 96.7% specificity, confirmed those of previous studies for serum MIC-1 in CRC, with minor differences in diagnostic sensitivity, possibly related to the patient characteristics [13, 18, 19]. Interestingly, Patients with advanced tumor and liver metastasis had substantially elevated levels of serum MIC-1 compared with those without liver metastasis. More than two third of our patients with distance metastasis had elevated concentrations, by contrast, 100% of the 11 recurrence patients with liver metastasis had abnormal MIC-1 serum concentrations, suggesting that CRC patients with liver metastasis may produce more MIC-1. No other reported studies have revealed this finding, but it seems reasonable in view of the highly presence of this protein in liver cancer tissues [22].

Though the molecular mechanism underlying the CRC's abnormalities is markedly improving which results in more targeted therapy and a decrease in cancer-related mortality recently, Carcinoembryonic antigen (CEA), still was used intensely but with varying results depending on the study design and the study population. CEA, which performed CRC screening on a serum level, had the notable advantage of simplicity and convenience in which it could be performed on a patient annually [8, 9]. Moreover, its lack of sensitivity in detecting early stage CRC made CEA determination especially poor for screening. In our study, we demonstrate the better MIC-1 diagnostic sensitivity and similar diagnostic specificity compared with CEA, and MIC-1 showed a similar sensitivity in advanced stages and higher sensitivity in early stages, revealing the higher serum MIC-1 utility in the early diagnosis of CRC. It is important to note that MIC-1 and CEA are related to tumor stage in colorectal cancer. These differences are mainly due to the early elevated concentrations of MIC-1 in stage I and II, and with no significant difference between stages III and IV. This advantage of MIC-1 seems to be greater in CRC screening, and need to be validated in studies with higher numbers of patients.

Various studies have suggested the serial use of serum CEA in combination with ultrasonography in asymptomatic subjects as an aid in the early diagnosis of CRC [23]. Problems with this strategy are related to the limited diagnostic sensitivity and specificity of serum CEA. In this study, we found that CEA and MIC-1 were complementary and their combined use could significantly increase the sensitivity obtained with either biomarker alone, primarily in early stage I–II. The panel with the MIC-1 and CEA achieved by the logistic regression model demonstrates high diagnostic accuracy (AUC=0.897; sensitivity=82%; specificity=89%) in differencing CRC from healthy controls. MIC-1 improves the utility of CEA as a tumor biomarker in CRC, and using both biomarkers simultaneously increases the sensitivity in CRC. It is therefore valuable to combine MIC-1 with clinically available biomarker CEA to discriminate normal tissue from CRC with high sensitivity without compromising specificity. We can conclude that MIC-1 is promising, noninvasive seromarkers and maybe a valuable supplements to the serum biomarkers already in use.

It is well-accept that screening programs are able to early detect and decrease mortality from CRC [20], while some will relapse in patients with CRC after underwent potentially curative resection [24–27]. Recurrences are mainly attributed to greater malignancy and poor response to chemotherapy, suggesting that a non-invasive blood based test with high sensitivity and specificity for monitoring the recurrences in patients with CRC will greatly attributed to higher survival. Moreover, Pathological staging based on the Tumor-Node-Metastasis (TNM) system is currently the major prognostic indicator for patients with CRC [28], which is, however, not accounting for the heterogeneity of individual tumors, there is an increasing demand for biomarkers that are involved specifically in CRC progression, thus facilitating a more accurate prognostic stratification of the tumor to improve efficacy of multimodal therapy. Hence, the predictive factors of early postoperative relapse with high sensitivity and specificity to precisely and reliably diagnose CRC and provide patient follow-up procedures are critical features that must be considered.

Survival of patients with CRC is highly associated with the clinical stage at diagnosis and metastasis status after treatment, especially liver metastasis [29]. Monitoring treatment response and tumor recurrence is another important role of tumor biomarker. In this research, surgical resection of CRC resulted in a decline in serum MIC-1 and the decreased serum MIC-1 was elevated at the presence of tumor recurrence. Moreover, our results indicated that measuring of serum MIC-1 after surgical treatment is helpful in prediction of cancer recurrence at early stage, especially with liver metastasis. This conclusion needs to be further addressed, as our patient sample number and the follow-up duration are not sufficient enough for this analysis; however, these results do represent preliminary evidence of a relationship between serum MIC-1 and CRC recurrence that warrants further exploration. Survival analysis indicated that patients with lower serum MIC-1 had a better prognosis in tumor-specific survival. Multivariate Cox analysis showed that serum MIC-1 is an independent prognostic indicator for CRC.

In summary, the current study provides further insight into the clinical value of MIC-1 by confirming that MIC-1 is complementary with CEA and involved in the development or recurrence of CRC with liver metastasis. Future research will be focused on the variation trend in serum MIC-1 that corresponding to every step of tumor development and progression so that we can further upgrade the diagnosis, monitoring, and prediction of CRC. But we also should remember, the wide range of MIC-1 serum concentrations found in patients with benign disease clearly indicated that serum MIC-1 should be interpreted cautiously in patients with inflammation [30]. This is important, since acute inflammation could be found in CRC patients during chemotherapy [31]. Despite these issues, serum MIC-1 is still valuably used for screening with CEA and as a prognostic indicator in malignant CRC, as well as for early assessment of recurrence with liver metastasis.

## MATERIALS AND METHODS

### Study population and study design

473 patients with CRC diagnosed between 2009 and 2011 from cancer institute and hospital, Chinese Academy of Medical Sciences (CICAMS), 25 patients with benign adenomatous polyp diagnosed by pathological examination and 489 healthy age- and gender-matched subjects were recruited by physical examination (Table 2). The clinicopathologic parameters of patients with CRC are summarized in Table 2. Samples from the patients with CRC and benign adenomatous polyp were obtained at diagnosis before any treatment, when they were admitted to the hospital. Moreover, serum samples from 106 of the 473 CRC patients undergoing clinical curative resection were collected at 4 weeks post-surgery, and 20 of the above 106 cases with relapsed disease were collected for detection of serum MIC-1 level in response to CRC recurrence. A total of 94 patients undergoing curative resection, with the average is 43 months and range is between 9-80 months, had follow-up information. Survival data were acquired from medical records and the research ends were recurrence as shown by medical imaging and patient's death from tumor-specific reasons. Deaths other than that were regarded as uncensored cases. The histopathological type and the clinical stage of cancer were in accordance with the criteria of the World Health Organization classification. The ethics approval was obtained from the Ethics Committee of CICAMS.

**Table 2:** Characteristics of subjects with CRC and controls

Variable	Serum samples (pre-operative)			Serum samples (post-operative)	Serum samples (relapse)
	Healthy controls (n = 489)	benign disease (n=25)	CRC Cases (n=473)	Cases(n= 106)	Cases(n= 20)
Gender(n)					
Male	265(54.2%)	11(44.0%)	295(62.4%)	69(66.3%)	14(70.0%)
Female	224(45.8%)	14(56.0%)	178(37.6%)	37(33.7%)	6(30.0%)
Age (years)					
≤60	279(57.1%)	11(44.0%)	247(52.1%)	65(61.3%)	8(40.0%)
>60	210(42.9%)	14(56.0%)	226(47.9%)	41(38.7%)	12(60.0%)
Stage (n)					
I			51(10.8%)	7(6.6%)	
II			153(32.3%)	38(35.8%)	6(20.0%)
III			201(42.5%)	47(44.3%)	12(60.0%)
IV			68(14.4%)	14(13.2%)	2(10.0%)

### Sample preparation and laboratory methods

Blood samples for CEA and MIC-1 analysis were collected by venous puncture in our hospital, centrifuged, and stored at -70°C before use. Serum were only thawed once just prior to experiment. Serum MIC-1 was detected with a sensitive in house sandwich ELISA, which was produced by CICAMS and described in our previous research in detail [32, 33]. All assays were repeated in duplicate. Serum CEA was detected by chemiluminescent enzyme immunoassay on an Architect® (Abbott Laboratories) by the use of a related kit (Roche). The cut-offs for CEA and MIC-1 was 5 U/L and 1000pg/mL, respectively.

### Statistical analysis

Data are presented herein as median (range). SPSS software (version 19.0) was employed for all the data analysis. Tumor biomarker concentrations between various groups were statistically compared by the Kruskall-Wallis and Mann-Whitney tests. The Wilcoxon test was used to compare paired samples. ROC curves were evaluated to determine the diagnostic efficacy of MIC-1 and CEA and compared by the DeLong mathematical model. Logistic regression model was binomial fitted to combine diagnostic performance of serum biomarkers. Parameters would be summarized and statistically analyzed to determine sensitivity, specificity, positive predictive value (PPV) and negative predictive value (NPV). Tumor-specific overall survival was analyzed by the Kaplan–Meier and Cox proportional hazard model. The level of statistical significance was set at a two-sided P < 0.05.
